# Liquid-Liquid Extraction of Indium(III) from the HCl Medium by Ionic Liquid A327H^+^Cl^−^ and Its Use in a Supported Liquid Membrane System

**DOI:** 10.3390/molecules25225238

**Published:** 2020-11-10

**Authors:** Francisco José Alguacil

**Affiliations:** Centro Nacional de Investigaciones Metalurgicas (CSIC), Avda, Gregorio del Amo 8, 28040 Madrid, Spain; fjalgua@cenim.csic.es

**Keywords:** A327H^+^Cl^−^ ionic liquid, extraction, membrane transport, indium, hydrochloric acid

## Abstract

Ionic liquid A327H^+^Cl^−^ was generated by reaction of tertiary amine A327 and HCl, and the liquid-liquid extraction of indium(III) from the HCl medium by this ionic liquid dissolved in Solvesso 100 was investigated. The extraction reaction is exothermic. The numerical analysis of indium distribution data suggests the formation of A327H^+^InCl_4_^−^ in the organic phase. The results derived from indium(III) extraction have been implemented in a supported liquid membrane system. The influence of the stirring speed (600–1200 min^−1^), carrier concentration (2.5–20% v/v) in the membrane phase, and indium concentration (0.01–0.2 g/L) in the feed phase on metal transport have been investigated.

## 1. Introduction

Indium is a metal with a sinusoidal life. In the 1960s, it had a high value, then practically disappeared from the market, and since the appearance of the “new technologies” its demand, usage, and price have rocketed. Effectively, indium is used (besides the indium and tin oxide (ITO) material which is the main product associated with the use of the metal) in energy-efficient windows, spectroelectrochemistry, organic light-emitting diodes, touch screens, flat screens, CD/DVD players, and solar panels, alloys of indium, ceramic materials, solders, as a catalyst in the benzylation reaction (in the form of rice husk ash supported indium), etc.

The main source of indium is zinc ores, thus, indium is a by-product of the zinc metallurgy; however, the interest has been discovered, and here, the concept of urban mining comes to mind, in the recycling of In-bearing materials, which include most of the cited above, but particularly, LCD screens and e-waste. The recovery of indium from such solid waste can be considered mainly via a pyrometallurgical or hydrometallurgical route [1]. However, pyrometallurgy, though of wide use, has a limited scope due to environmental drawbacks; hydrometallurgy is more targeted than pyrometallurgy due to various advantages, such as easy to control chemical reaction, less intensive, non-specific process parameters, fewer emissions, low energy input, etc. [2]. Hydrometallurgical processes, such as acid leaching, alkali leaching, bioleaching, solvent extraction, electrometallurgy, super- and sub-critical fluid extractions, precipitation and cementation have gained much interest in the recovery and recycling of indium.

Hydrometallurgical recovery of indium from a material containing it requires some steps: the first is to leach the solid product, and in the case of indium, as expected, mineral acids and aqua regia is the media to dissolve the element [3,4], though incursions in the use of bioleaching [5] and deep eutectic solvents [6] are also known. Once dissolved, the approaches to recover the metal include ion exchange [7], precipitation [8], membranes [9,10], counter-current foam separation [11], electrowinning [12], cementation [4], but the main interest seemed to be in the use of solvent extraction using conventional extractants, such as 8-hydroxyquinoline derivatives [13], D2EHPA [14], TBP (tributyl phosphate [15], methylimino-dioctylacetamide (MIDOA) [16], ionic liquids (Cyphos IL101 and Aliquat 336 [17], Cyphos IL104 [18], A324H^+^Cl^−^ [19], PJMTH^+^HSO_4_^−^ [20]), or chloride-rich deep eutectic solvents [21]; in all the above cases, the extraction efficiencies of the different extractants were high, i.e., exceeding 95%, though the experimental conditions vary from one investigation to another, i.e., pH 2 [13] against a medium rich in HCl [15,19], a type of an acidic medium, i.e., HCl [16] against sulphuric acid [20], and varying extractant concentrations [13,14,15,16,17,18,19,20,21].

Ionic liquids are a group of chemicals, which are considered of interest in the field of liquid–liquid extraction due to the ability to react with metal compounds [22,23,24,25], and also due to their properties, such as low melting point, nonflammability, high thermal stability, and low vapor pressure; due to their non-volatile nature, they are called “green solvents”, though there are voices claiming about their harmfulness and the toxicity associated with some of their components. 

Considering liquid membrane technologies and a specially supported liquid membrane operation mode, the interest of the technology in the processing of metals-bearing aqueous solutions is in their operational characteristics, which include, among other things, the low energy input associated with the technology, the possibility of treatment of metal-diluted solutions [26,27], as well as the possibility of using the same type of extractants, including ionic liquids, as in the case of conventional liquid-liquid extraction operation.

The present investigation is undertaken to derive a quantitative characterization of the extraction reactions between indium(III) in an HCl medium and an A327H^+^Cl^−^ ionic liquid dissolved in Solvesso 100. In addition, a supported liquid membrane system was investigated by using the extraction information mentioned above and different parameters affecting metal transport, i.e., stirring speed of the feed phase, composition of the membrane phase, and metal concentration in the feed phase were investigated.

## 2. Results and Discussion

### 2.1. Generation of Ionic Liquids

**Figure 1:**
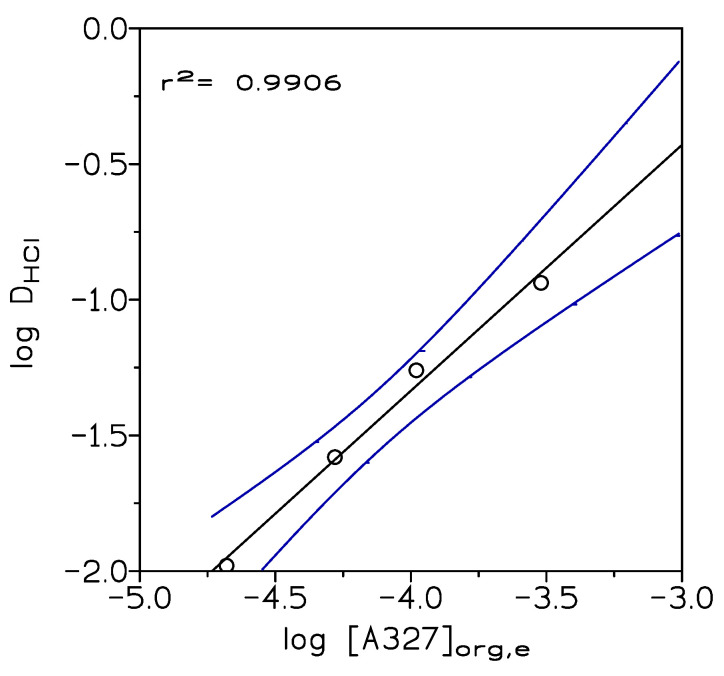
Hydrochloric acid extraction by A327 solutions (0.5–5% v/v) in Solvesso 100. Temperature: 20 °C. Equilibration time: 10 min. Organic/Aqueous phases relationship: 1. Dotted line showed the 95% confidence interval of the regression line.

This type of ionic liquids, a form of quaternary ammonium salts R_4_N^+^Cl^−^, can be generated by reaction of a given amine and the corresponding mineral acid [28,29]. Figure 1 shows the results of extraction of 1 M HCl solution by different amine concentrations in Solvesso 100; it can be seen that a straight line with a slope near 1 was obtained; thus, the ionic liquid was formed according to:

(1) $$A327_{org}+H_{aq}^{+}+Cl_{aq}^{-}\Leftrightarrow A327H^{+}Cl_{org}^{-}$$

with log K = 2.29 (graphically obtained). The experimental data were numerically treated by a computer program which minimizes function U:

(2) $$U=\Sigma {\left(\log D_{cal}-\log D_{\exp}\right)}^{2}$$

where D_cal_ and D_exp_ were the calculated and the experimental distribution coefficients values, respectively.

The numerical treatment proposed formation in the organic phase of a liquid, that is, an ionic liquid, with the stoichiometry indicated in Equation (1) and log K = 2.55 (U = 3.8 × 10^−6^, σ(log K= 0.558)). In the present study, the ionic liquid was generated in Solvesso 100; this procedure has the advantage of (i) decreasing the viscosity of the ionic liquid and thus of improving phase disengagement, (ii) adjusting the extractant concentration to a given system, avoiding the use of unnecessary extractant concentration excess, and (iii) the yield of an organic phase containing the ionic liquid ready for its further use in metal extraction processing.

### 2.2. Indium(III) Liquid-Liquid Extraction Results

To investigate the effect of the equilibration time on indium(III) extraction by a A327H^+^Cl^−^ ionic liquid, various extractions were carried out using O/A relationships of 1 and an organic phase of 1.25% v/v ionic liquid in Solvesso 100 and an aqueous solution containing 0.075 g/L In(III) in 7 M HCl. The temperature was 20 °C and the equilibration time varied from 1 to 60 min. The results show that, under the present experimental conditions, the extraction is not very dependent upon the contact time and equilibrium is achieved after 5 min of contact (94% indium extraction, equivalent to an indium distribution coefficient value of 15.7).

The variation of the temperature (20–60 °C) in the extraction of indium(III) by an ionic liquid was investigated using the same organic and aqueous solutions as described above. The results given in Table 1 indicated that there is a decrease in indium extraction as the temperature is increased, the extraction reaction is exothermic with ΔH° = −15 kJ/mol.

The metal distribution ratio at 7 M HCl was determined for different indium(III) concentrations. The results are showed in Table 2. It can be seen that D_In_ does not depend on the indium concentration. This behavior indicates that metal-polynuclear complexes are not apparently formed in the organic phase.

The variation in the concentration of the ionic liquid in Solvesso 100 in the extraction of indium(III) was also investigated. The temperature was 20 °C, and the aqueous solution contained 0.075 g/L In(III) in 7 M HCl, with the results derived from this investigation showed in Table 3.

It can be clearly seen that a ten-fold increase of the extractant concentration in the organic phase increased the indium(III) distribution coefficient value nearly 150 times. 

Accordingly with the literature [30], at this HCl concentration in the aqueous solution, indium(III) is present in this solution as InCl_4_^−^ species, thus, the extraction of indium(III) can be described by using the following formula:

(3) $$InCl_{4_{aq}}^{-}+A327H^{+}C{l^{-}}_{org}\Leftrightarrow A327H^{+}InCl_{4_{org}}^{-}+Cl_{aq}^{-}$$

which is in accordance with the previous assumption that non-polynuclear metal complexes are formed in the organic phase.

Considering this equilibrium, experimental data were treated numerically by a program which minimizes the U function, as described in Equation (1). The results indicated the existence in the organic phase of the ionic liquid In(III) species showed in Equation (3), with log K = 3.74 (U = 0.7889, σ(log K = 0.482)). Thus, the extraction of indium(III) from this 7 M HCl medium matched an anionic exchange reaction.

**Figure 2:**
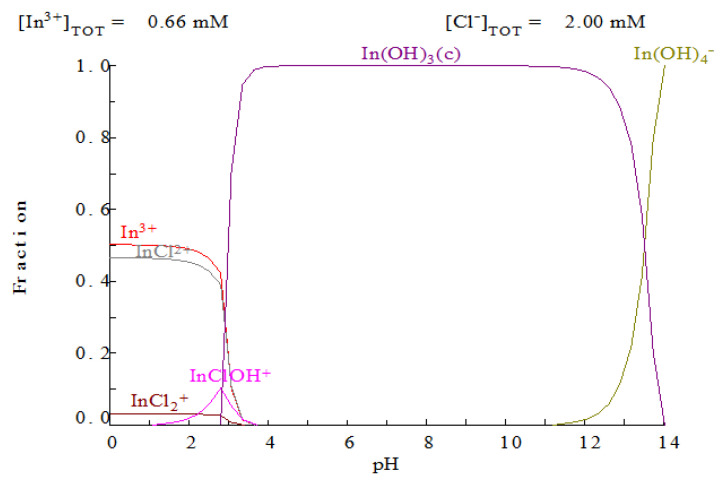
Indium(III) distribution depending on the pH function [31].

Indium(III) stripping from metal-loaded organic phases was also investigated. Considering that at low chloride concentrations, indium(III) exists in aqueous solutions as In^3+^ (Figure 2), it is logical to use low acidic solutions as a strippant for this system.

In this study, a 5% v/v A327H^+^Cl^−^ in Solvesso 100 loaded with 0.074 g/L indium(III) and 0.1 M HCl as a strip solution were used. The results of this study were summarized in Table 4; it can be seen that the percentage of indium(III) stripped, calculated as:

(4) $$\%St=\frac{{\left[\mathrm{In}\right]}_{\mathrm{st}}}{{\left[\mathrm{In}\right]}_{\mathrm{org}}}\times 100$$

decreased as the Organic/Strip phases relationship increased. In the above equation, [In]_st_ and [In]_org_ were the indium concentrations in the equilibrated strip solution and in the organic phase feeding the stripping stage, respectively. In these series of experiments, equilibrium was reached within 5 min of contact between both phases.

Accordingly with these results, the stripping reaction can be written as:

(5) $$A327H^{+}\mathrm{InC}l_{4_{\mathrm{org}}}^{-}\Leftrightarrow A327H^{+}Cl_{\mathrm{org}}^{-}+In_{aq}^{3+}+3Cl_{aq}^{-}$$

and:

(6) $$A327H^{+}\mathrm{InC}l_{4_{\mathrm{org}}}^{-}\Leftrightarrow A327H^{+}Cl_{\mathrm{org}}^{-}+\mathrm{InC}l_{\mathrm{aq}}^{2+}+2Cl_{\mathrm{aq}}^{-}$$

### 2.3. Indium(III) Transport Results

The system In(III)/A327H^+^Cl^−^ was also investigated in a supported liquid membrane; in this operation, the transport of indium(III) across the liquid membrane also depends on kinetic factors.

The influence of the stirring speed applied to the feed phase on In(III) transport was first investigated, with the results derived from these tests summarized in Table 5.

The permeability coefficient increased from 1.0–1.4 × 10^−1^ cm/min in the range of stirring speeds from 600 to 850 min^−1^, and beyond that, no appreciable increase in indium(III) transport was observed. These results indicated that the thickness of the feed phase boundary layer decreased continuously with the increase of the stirring speed, and that the boundary layer reached the minimum in the 850–1000 min^−1^ range. A slight decrease in permeability at 1200 min^−1^ could be attributed to an increment in the turbulence caused by stirring resulting in displacement of the carrier phase from the support pores. A stirring speed of 865 min^−1^ in the feed phase was maintained throughout all the experimentation.

Using the same experimental conditions as in Table 5, the variation of the stirring speed (500–750 min^−1^) in the receiving solution showed no changes in the permeability coefficient. Thus, a stirring speed of 500 min^−1^ in the receiving solution was used in all the experiments. Furthermore, the variation in the receiving solution composition in the indium(III) transport was investigated. In this case, all the experimental parameters were maintained constant as in Table 5, but the receiving phase was of 0.01 g/L In(III) and 0.1 M HCl. The results derived from this experiment showed that the presence of indium(III) in the initial receiving phase practically did not change indium permeability: 1.5 × 10^−1^ cm/min for the present receiving solution against 1.4 × 10^−1^ cm/min in the case of the receiving solution containing only 0.1 M HCl. However, the importance of this experiment was that it demonstrated that indium(III) is transported against its concentration gradient for the first 15 min, driven by the difference in acidity (or chloride concentration) between the feed and the receiving phases (Table 6).

Moreover, if the chloride concentration in the feed phase, from the HCl medium, was substituted by LiCl, the transport experiment would allowed concluding that this substitution resulted in a decrease in indium transport (P = 5.9 × 10^−2^ cm/min). Thus, the difference in acidity between the feed and the receiving phases was the main driving force for this system.

The variation (0.01–0.2 g/L) in indium(III) concentration in the feed solution in the metal transport was also investigated, with the results obtained from these tests shown in Table 7.

In this Table 7, the initial metal flux J was calculated as:

(7) $$J=P_{\mathrm{In}}{\left[\mathrm{In}\right]}_{0}$$

where [In]_0_ represented the initial indium concentration in the feed phase. The percentage of indium recovered in the receiving solution (%R) was calculated by the next relationship:

(8) $$\%R=\frac{{\left[\mathrm{In}\right]}_{r,t}}{{\left[\mathrm{In}\right]}_{0}-{\left[\mathrm{In}\right]}_{f,t}}\times 100$$

where [In]_f,t_ was the metal concentration in the feed solution at a specific timepoint and [In]_r,t_ was the indium concentration in the receiving phase at the same timepoint.

At these initial indium(III) concentrations in the feed phase, the initial flux was a function of the initial metal concentration in the phase. Therefore, the transport process was controlled by diffusion of indium species. With respect to the recovery of the metal in the receiving phase, acceptable values were obtained in all the cases, though these values could be improved by the use of the smart liquid membrane technology such as pseudo-emulsion membrane strip dispersion [32]. 

**Figure 3:**
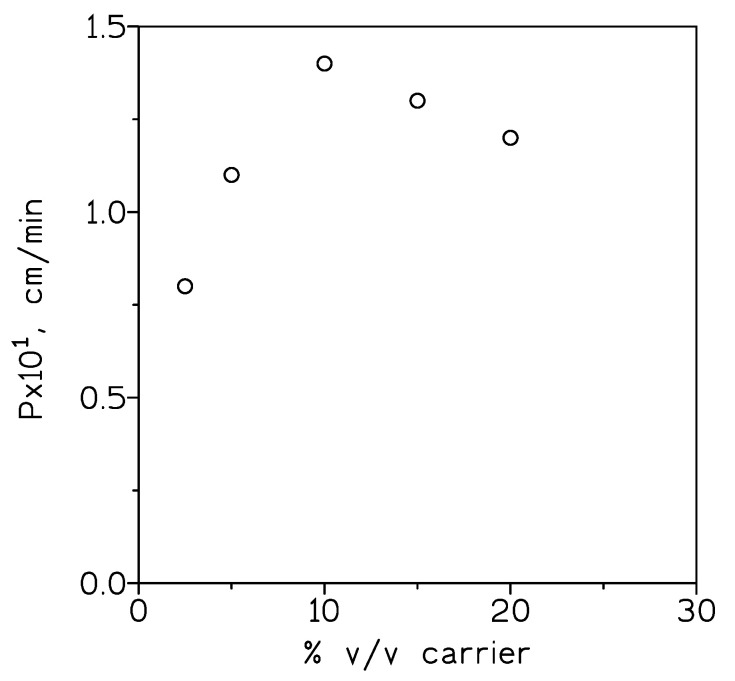
Indium(III) permeability versus carrier concentration. Feed phase: 0.01 g/L In(III) and 7 M HCl. Stirring speed: 865 min^−1^. Membrane phase: carrier solutions in Solvesso 100 immobilized in the GVHP4700 support. Receiving phase: 0.1 M HCl. Stirring speed: 500 min^−1^. Temperature: 20 °C.

The effect of the carrier concentration on indium(III) transport was also investigated. Figure 3 showed the permeability values for the transport of indium(III) across a supported liquid membrane containing different solutions of the ionic liquid in Solvesso 100. The results showed that the permeability increased with the A327H^+^Cl^−^ concentration until the maximum value of P was obtained at the 10% v/v carrier concentration. This limiting permeability value, P_lim_, can be explained by assuming that diffusion across the membrane was negligible compared with that of aqueous diffusion, with the transport process controlled by the diffusion of metal species in the stagnant layer of the feed phase. In that case,

(9) $$P_{\lim}=\frac{D_{\mathrm{aq}}}{d_{\mathrm{aq}}}$$

where D_aq_ was the average aqueous diffusion coefficient (6 × 10^−4^ cm^2^/min) and d_aq_ was the thickness of the feed phase diffusion layer, thus, P_lim_ = 1.4 × 10^−1^ cm/min, the value of d_aq_ was 4 × 10^−3^ cm; this value represented the minimum thickness of the diffusion layer. The decrease in the permeability value at higher carrier concentrations was probably due to an increase in viscosity of the organic solution, which caused an increase in membrane resistance to indium(III) transport.

At the first approach, the mechanism of mass transfer in flat sheet supported liquid membranes is understodd as the diffusion of the indium species across the membrane pores from the feed phase to the receiving phase with no dispersion at all. Evaluation of mass transfer coefficients is important, because they influence the scaling-up of the technology to the most efficient membrane operation using a hollow fibers module, which gives an important increment in the contact area between the feed-organic phases and the organic–receiving phases. According to Equation (3), the equilibrium constant for the present system can be written as:

(10) $$K=\frac{{\left[A327H^{+}\mathrm{InC}l_{4}^{-}\right]}_{\mathrm{org}}{\left[Cl^{-}\right]}_{\mathrm{aq}}}{{\left[A327H^{+}Cl^{-}\right]}_{\mathrm{org}}{\left[\mathrm{InC}l_{4}^{-}\right]}_{\mathrm{aq}}}$$

Following the same approach as described elsewhere [33], the next expression can be derived:

(11) $$P_{\mathrm{In}}=\frac{K{\left[A327H^{+}Cl^{-}\right]}_{\mathrm{org}}{\left[Cl^{-}\right]}_{f}^{-1}}{\Delta_{m}+\Delta_{f}\left(K{\left[Cl^{-}\right]}_{f}^{-1}{\left[A327H^{+}Cl^{-}\right]}_{\mathrm{org}}\right)}$$

where Δ_f_ and Δ_m_ are the transport resistances related to diffusion in the feed and membrane phases, respectively. In the above equation, P_In_ is the indium(III) permeation coefficient, moreover, Equation (11) combines the diffusional and equilibrium parameters involved in the transport of indium(III) from an HCl solution through a membrane containing the carrier. From the above,

(12) $$\frac{1}{P_{\mathrm{In}}}=\Delta_{f}+\Delta_{m}\frac{1}{K{\left[Cl^{-}\right]}_{f}{\left[A327H^{+}Cl^{-}\right]}_{\mathrm{org}}}=\Delta_{f}+\frac{\Delta_{m}}{B}$$

Accordingly, a plot of 1/P_In_ versus 1/B for various carrier concentrations in the membrane phase and 7 M chloride concentration in the feed phase resulted in a straight line (r^2^ = 0.9115) with an intercept and a slope that can be used to estimate the transport resistance in the feed phase (Δ_f_ = 7 min/cm) and in the membrane phase (Δ_m_ = 170 min/cm), respectively. 

Thus, the mass transfer coefficient in the feed phase is Δ_f_^−1^ = 1.4 × 10^−1^ cm/min, whereas the diffusion coefficient in the organic solution,

(13) $$D_{\mathrm{org}}=\frac{d_{m}}{\Delta_{m}}$$

is calculated as 7.4 × 10^−5^ cm^2^/min, considering Δ_m_ is 170 min/cm and the thickness of the membrane support, d_m_, is 12.5 × 10^−3^ cm.

The diffusion coefficient of the indium(III) ionic liquid species in the bulk organic phase is estimated using the following relationship [34]:

(14) $$D_{\mathrm{org}}=D_{b,\mathrm{org}}\frac{\varepsilon }{\tau^{2}}$$

where ε is the support porosity (75%) and τ is the membrane tortuosity (1.67), thus, D_b,org_ is estimated at 2.8 × 10^−4^ cm^2^/min. The diffusion coefficient in the bulk organic phase presented a greater value than D_org_; this result can be due to the diffusional resistance caused by support thickness located between the feed and receiving solutions.

If the carrier concentration in the membrane support is constant, the apparent diffusion coefficient for indium(III) can be calculated as:

(15) $$D_{m}^{a}=\frac{Jd_{m}}{\left[\mathrm{carrier}\right]}$$

Using a 10% v/v ionic liquid concentration (2.1 × 10^−1^ M) and with d_m_ = 12.5 × 10^−3^ cm, this apparent diffusion coefficient has the value of 7 × 10^−7^ cm^2^/min.

## 3. Materials and Methods

The source for the generation of the ionic liquid was HCl and tertiary amine A327 (Sanofi), which consisted of a 50% mixture of tri-octyl and tri-decyl amines, with the average molecular weight of 395 and the density of 0.82 g/cm^3^ (20 °C); the reagent was diluted in Solvesso 100 (ExxonMobil Chem., Iberia, Spain) having > 99% aromatics. All other chemicals used in this work were of the AR grade. The aqueous solutions used in the liquid-liquid extraction and membrane investigations were prepared using a stock indium(III) solution. HCl concentration was kept constant at 7 M in all the experiments, as previous data demonstrated that the maximum indium extraction was yielded using this HCl concentration.

### 3.1. Liquid-Liquid Extraction Experiments 

Generation of an ionic liquid: extraction experiments were carried out at 20 °C by mechanical shaking (four-blades glass impeller of 2.5 cm diameter) of equal volumes (25 cm^3^) of the aqueous and organic solutions in separatory funnels for the required time. After phase separation (always less than 2 min), the HCl concentrations in the equilibrated organic and aqueous phases were determined by titration with standard NaOH solutions using bromothymol blue as an indicator. The results were expressed as the HCl distribution coefficient defined as follows:

(16) $$D_{\mathrm{HCl}}=\frac{{\left[\mathrm{HCl}\right]}_{\mathrm{org},e}}{{\left[\mathrm{HCl}\right]}_{\mathrm{aq},e}}$$

Indium extraction and stripping: experiments were carried out in the same form and device as described above, and the metal remaining in the raffinate (or in the aqueous strip solution) was analyzed by AAS (Perkin Elmer 1100B spectrophotometer, Cambridge UK). The indium concentration in the organic phase was obtained by difference with the initial concentration in the aqueous phase. The associated error with this procedure was ±5%. As above, the results were expresses as follows:

(17) $$D_{\mathrm{In}}=\frac{{\left[\mathrm{In}\right]}_{\mathrm{org},e}}{{\left[\mathrm{In}\right]}_{\mathrm{aq},e}}$$

where [In]_org,e_ and [In]_aq,e_ are the indium concentrations in the equilibrated organic and aqueous phases, respectively.

### 3.2. Supported Liquid Membrane Experiments

These experiments were carried out in a permeation cell consisting of two half-cells (200 cm^3^ each) made of methacrylate and separated with a solid support. The feed solution contained indium(III) in 7 M HCl, whereas the receiving solution was 0.1 M HCl. The solid support was Durapore GVHP4700 (Millipore, Tullagreen, County Cork, Ireland) with a thickness of 125 μm, porosity of 75%, and the average pore size of 0.22 μm. The liquid membrane was prepared by impregnation of the support in the corresponding organic solution for 24 h.

The indium content in both the feed and the receiving phases was periodically determined as above. The permeability coefficient (P_In_) was calculated using the slope of the next expression:

(18) $$\ln\frac{{\left[\mathrm{In}\right]}_{f,t}}{{\left[\mathrm{In}\right]}_{f,0}}=-\frac{AP_{\mathrm{In}}}{V}t$$

where [In]_f,0_ and [In]_f,t_ are the indium concentrations in the initial feed solution and in the same phase at a specific timepoint, respectively, A is the membrane area (11.3 cm^2^), V is the volume of the feed phase (200 cm^3^), and t is time.

## 4. Conclusions

Ionic liquid A327H^+^Cl^−^ is generated by reaction between tertiary amine A327 and hydrochloric acid; this ionic liquid dissolved in Solvesso 100 can extract indium(III) from a 7 M HCl medium. There is a decrease in metal extraction with the increase of the temperature, so the extraction is exothermic. Indium extraction is dependent on the extractant concentration, but it is independent of the initial metal concentration in the aqueous solution. Indium(III) extraction by this ionic liquid can be associated with an anion exchange reaction, with formation of an A327H^+^InCl_4_^−^ species in the organic phase. Indium can be stripped from loaded organic phases by the use of low acidic solutions, i.e., 0.1 M HCl.

The extraction system has been implemented in a supported liquid membrane process in which, and under the present experimental conditions, indium flux is dependent on the initial metal concentration of the feed phase. Metal permeation is dependent on carrier concentration, though for a carrier concentration of 10% v/v in Solvesso 100, a limiting permeability value is obtained, and in this condition, the transport process is controlled by the diffusion in the feed phase stagnant layer; outside of this limiting condition, membrane diffusion controls the overall indium(III) transport.

## Figures and Tables

**Table 1:** Variation of indium extraction with temperature.

Temperature, °C	D_In_
20	15.7
30	13.3
40	11.5
50	9.0
60	7.3

Equilibration time: 10 min. O/A relationship: 1.

**Table 2:** Experimental distribution data at various metal concentrations.

[In]_0_, g/L	D_In_
0.010	15.7
0.075	15.7
0.15	15.9

Organic phase: 1.25% v/v ionic liquid in Solvesso 100. Temperature: 20 °C. Equilibration time: 10 min. O/A relationship: 1.

**Table 3:** Influence of the extractant concentration on In(III) extraction.

Ionic Liquid Concentration, % v/v	D_In_
5	500
2.5	40.7
1.25	15.7
0.50	3.3

O/A relationship: 1.

**Table 4:** Indium(III) stripping at various O/A relationships.

O/A	%St
0.5	99
1	79
2	64
3	45
4	36

Temperature: 20 °C. Equilibration time: 10 min.

**Table 5:** Influence of the stirring speed on In(III) permeability.

Stirring Speed, min^−1^	P_In_ × 10^1^, cm/min
600	1.0
850	1.4
1000	1.4
1200	1.2

Feed phase: 0.01 g/L In(III) and 7 M HCl. Membrane phase: a 10% v/v ionic liquid in Solvesso 100 immobilized in the GVHP4700 support. Receiving phase: 0.1 M HCl. Stirring speed: 500 min^−1^. Temperature: 20 °C.

**Table 6:** Indium concentrations in the feed and receiving phases at different timepoints.

Time, min	[In]_f,t_, mg/L	[In]_r,t_, mg/L
0	10.0	10.0
15	8.9	11.0
30	7.8	12.0
45	6.7	13.2
60	6.1	13.8
120	3.7	15.9
180	2.3	16.7

Feed phase: 0.01 g/L In(III) and 7 M HCl. Stirring speed: 865 min^−1^. Membrane phase: a 10% v/v ionic liquid in Solvesso 100 immobilized in the GVHP4700 support. Receiving phase: 0.01 g/L In(III) and 0.1 M HCl. Stirring speed: 500 min^−1^. Temperature: 20 °C.

**Table 7:** Influence of the initial In(III) concentration in the feed phase on metal transport.

[In]_0_, g/L	P_In_ × 10^1^, cm/min	J × 10^7^, mol/cm^2^ min	%R ^a^
0.01	1.4	0.12	85
0.05	1.3	0.57	89
0.1	1.1	0.96	96
0.2	0.94	1.6	90

Feed phase: In(III) and 7 M HCl. Stirring speed: 865 min^−1^. Membrane phase: a 10% v/v ionic liquid in Solvesso 100 immobilized in the GVHP4700 support. Receiving phase: 0.1 M HCl. Stirring speed: 500 min^−1^. Temperature: 20 °C. ^a^ After 3 h.
